# Endosymbiotic Bacteria *Spiroplasma* and *Wolbachia* in a Laboratory-Reared Insect Collection

**DOI:** 10.3390/insects16111168

**Published:** 2025-11-16

**Authors:** Roman Bykov, Elena Shatalova, Irina Andreeva, Alevtina Khodakova, Artem Ryabinin, Mary Demenkova, Yury Ilinsky

**Affiliations:** 1 Institute of Cytology and Genetics, Siberian Branch of Russian Academy of Sciences, 630090 Novosibirsk, Russia; bykovra@bionet.nsc.ru (R.B.); art@bionet.nsc.ru (A.R.); judina@bionet.nsc.ru (M.D.); 2 Siberian Federal Scientific Centre of Agro-BioTechnologies of the Russian Academy of Sciences (SFSCA RAS), 630501 Krasnoobsk, Russia; elenashatalova@mail.ru (E.S.); ivaandreva@yandex.ru (I.A.); khodakova.alevtina@bk.ru (A.K.)

**Keywords:** symbiosis, arthropods, *Chrysopa formosa*, *Macrolophus pygmaeus*, *Nabis* sp., *Tenebrio molitor*, *Trissolcus kozlovi*

## Abstract

Many insect and other arthropod species are maintained as non-model laboratory stocks and are used for fundamental and applied studies. Their biology may be affected by symbionts, such as *Wolbachia* and *Spiroplasma*. Thirty stocks of different insect species that are maintained at the Laboratory of biological control of phytophagous and phytopathogens in the Siberian Federal Scientific Centre of Agro-BioTechnologies were screened to find *Spiroplasma*/*Wolbachia*–host associations. We used *16S* rDNA and *fusA* loci for *Spiroplasma* characterization and five MLST genes for *Wolbachia*. Seven out of thirty stocks harbored symbionts. Five stocks were infected with only *Wolbachia*, one with only *Spiroplasma*, and one with both symbionts. Two stocks were occasionally characterized by false-positive signals of *Spiroplasma* infection that were explained by contamination from food sources, viz. infected insects. Five *Wolbachia* isolates belonged to supergroup B and one to supergroup A. Only the MLST haplotype of *Nabis ferus* was previously known (ST-522), while the other haplotypes contained new alleles. One *Spiroplasma* isolate was clustered in the Ixodetis clade and another was basal to the Apis clade. We noted the importance of non-model insects for fundamental studies of host–symbiont interactions and their significance for applied research and practice.

## Simple Summary

Endosymbiotic bacteria, such as *Wolbachia* and *Spiroplasma*, can significantly influence the biology of host species. Therefore, it is essential to consider their presence and influence when working with laboratory insect cultures. We studied laboratory-reared insect stocks of non-model species for *Spiroplasma* and *Wolbachia* symbionts. Out of the thirty stocks, seven contained symbionts: five species had only *Wolbachia*, one had only *Spiroplasma*, and one carried both. We provided genotyping of the symbiont isolates and discussed the fact that laboratory non-model insects are an important source for studying host–symbiont interactions and that our findings can also be used for practical applications.

## 1. Introduction

Many insect and other arthropod species are used for fundamental and applied studies. To maintain a non-model species stock, researchers collect insect specimens in the field, maintain a primary laboratory population, and characterize the population for morphology, physiology, and genetics. They also estimate it for special traits such as sex ratio, fertility, duration of development, reproductive and pre-reproductive periods, as well as the range of abiotic conditions for rearing. These traits and indicators can be affected by symbionts. Here, we have screened an insect stock collection, mainly non-model insect species, for *Spiroplasma* and *Wolbachia* symbionts. These bacteria are deeply integrated into the biology of their host species and can influence the physiological traits [1,2,3]. Characteristics of infection status and symbiont genetics of particular insect stocks can potentially be used in fundamental studies of host–symbiont interactions and their mechanisms and for applied research to optimize rearing. To share possible perspectives on such knowledge, we briefly review the biology of these symbionts and their influence on hosts.

*Wolbachia* are the most widespread symbiotic bacteria in arthropods, infecting more than 60% of arthropod species and some nematode species [4,5,6,7,8]. These intracellular symbionts are transmitted mostly vertically, from mother to offspring [1]. However, there is evidence of horizontal transmission of *Wolbachia* between host species [9,10,11]. According to phylogenetic reconstructions, there are at least 21 *Wolbachia* clades that are called supergroups. Strains of the A and B supergroups predominate in insect species [12,13,14,15,16,17,18,19,20,21]. Bacteria *Spiroplasma* are also widespread in various arthropods, infecting plants, and are even found in vertebrates including mammals [22,23]. *Spiroplasma* belong to the Mollicutes class, which also includes pathogens such as *Mycoplasma*, *Entomoplasma*, *Phytoplasma*, and others. There are two modes of spiroplasmas transmission in hosts: vertical (from mother to offspring) and horizontal (via common substrates) [2]. More than 40 species of *Spiroplasma* are described and they are subdivided into several phylogenetic clades: Apis, Citri–Chrysopicola–Mirum (CCM) and Ixodetis [24,25]. The latter is the most widespread in arthropods [26].

Both *Wolbachia* and *Spiroplasma* may have a wide range of effects on their hosts. In particular, both bacteria induce male killing and cytoplasmic incompatibility in different insect species [27,28,29,30,31,32,33,34]. *Wolbachia* also induce feminization and thelytokous parthenogenesis [32]. Besides these reproductive abnormalities, some mutualistic effects of the symbionts, such as suppression of mutations, increased fitness and longevity for *Wolbachia* [35,36,37,38], and protection from parasitoids, nematodes, and fungi for both bacteria [39,40,41,42] are reported. Some *Spiroplasma* species are pathogens of plants, arthropods, and vertebrates [43,44,45]. Co-infection with *Wolbachia* and *Spiroplasma* is shown for a spider mite *Tetranychus truncatus* [46,47], where the symbionts alter the expression of many genes, increase female fecundity and male hatchability, and accelerate development, but also induce cytoplasmic incompatibility [46]. In addition, co-infection alters host plant defense, which is manifested in decreased expression of jasmonic and salicylic acid responsive genes [48].

Current questions in studies of these bacteria are regarding their effects on hosts and the possibility of their use for practical purposes, e.g., for increasing host fecundity and defending it from environmental factors. Another question concerns the mechanisms of the symbiont–host interaction based on new model systems. In the present study, we aim to find potential new models of *Spiroplasma*/*Wolbachia*–host associations that can be used for fundamental and applied research.

## 2. Material and Methods

### 2.1. Insect Collection

Thirty insect stocks were used in this study (Table 1). All stocks were maintained at the Laboratory of biological control of phytophagous and phytopathogens SFSCA RAS, Krasnoobsk, Russia (collection supported by the FNUU-2024-0002). The origin of most stocks was field collections in the forest–steppe zone of Western Siberia. Insects were collected using standard entomological methods. Some stocks were established based on the material provided by other researchers (private stocks). The *Macrolophus pygmaeus* stock was founded from specimens collected in greenhouse farming in Novosibirsk Province (Russia). All stocks were maintained independently without crosses with any other stocks. Species identification was performed according to morphological traits using keys in the relevant guides [49,50,51,52]. The stocks were kept in plastic boxes (30 cm × 40 cm × 25 cm) or in cages (40 cm × 40 cm × 40 cm) covered with tulle. Environmental conditions for insect maintenance were as follows: temperature range of 24–27 °C, humidity of 30–60%, and lighting for no less than 12 h per day. The insects were fed 2–3 times per week with the appropriate nutrient substrate (Table 1) [53,54,55].

### 2.2. DNA Extraction

All insect specimens were frozen at −20 °C prior to DNA extraction. Depending on specimen size, we used part of an insect, a whole insect, or a pool of insects. In particular, for insects larger than 15 mm (*Acheta domesticus*, *Arma custos*, *G. bimaculatus*, *L. oleracea*, *Platymeris biguttatus*, *Psytalla horrida*) and for all Coccinellidae, an abdomen or gut and reproductive tissues were used because the reproductive and digestive systems were the focus of the *Wolbachia* and *Spiroplasma* analysis (Table S1). The whole insect was used in the case of specimens in the range of 5–15 mm (*B. rufimanus*, *C. formosa*, *M. pygmaeus*, *Nabis* species, *Nesidiocoris tenuis*, *P. maculiventris*). A pool of 5–10 specimens of insects smaller than 5 mm was used for DNA extraction (*Trissolcus kozlovi*, *Trialeurodes vaporariorum*, aphids). DNA extraction was repeated from different generations of stocks. In addition to extraction of adult specimens, we also used eggs, larvae, and nymphs in some cases. Material for extraction was homogenized in 200–400 µL of extraction buffer (10 mM Tris-HCl at pH 8.0, 25 mM EDTA, 0.5% of SDS, and 0.1 M NaCl) depending on specimen size and incubated at 56 °C for 1–2 h. After precipitation, DNA was dissolved in 100–200 µL deionized water. The quality of DNA samples was estimated using a NanoDrop One spectrophotometer (Thermo Fisher Scientific, Waltham, MA, USA) and by amplification with primer set LCO1490/HCO2198 [56].

### 2.3. PCR and Sequencing

For *Wolbachia* screening, we used two loci; in particular, we applied a nested PCR approach for the *ftsZ* gene and conventional PCR for the *coxA* gene. In the first round of nested PCR, the primer set ftsZunif/ftsZunir [14] was used, and in the second round, ftsZF1/ftsZR1 primers [57] were used. For the *coxA* gene, the primer set coxAF1/R1 [57] was used. The Bi90 strain of *Drosophila melanogaster* [58] was used as a positive control for *Wolbachia* infection and sterile water as a negative control. Screening for *Spiroplasma* infection was performed using the primer set SpiF1/SpiR3 [59] for the *16S* rDNA locus (fragment size of approximately 1067 bp). An infected sample of *Tabanidae* sp. was used as a positive control and sterile water as a negative control. *Wolbachia*-positive samples were characterized using five loci of the MLST protocol [57]. *Spiroplasma*-positive samples were sequenced using the above-mentioned *16S* rDNA primers. In addition, we elaborated a primer set for the elongation factor G (*fusA*) gene. According to our analysis, *fusA* is a core gene of *Spiroplasma* genomes with relatively conserved regions, which allowed us to design the following oligonucleotides: SfusAF 5′-CACGTTGAYTTYACWGTTGAAGT-3′ and SfusAR 5′-CCAACAAAWGGGTCWGTCAT-3′. This primer set produces an approximately 700 bp amplicon. Infected insect stocks were characterized using the barcoding region of the *co1* gene with primer set LCO1490/HCO2190.

PCR cycling conditions were 95 °C for 5 min, followed by 35 cycles at 95 °C for 15 s for *co1* and *16S* rDNA and 10 s for *fusA*, 53 °C for 1 min for *co1*, 40 s for *16S* rDNA, 55 °C for 30 s for *fusA*, 72 °C for 3 min for *co1*, 50 s for *16S* rDNA and *fusA*, plus a final elongation step of 72 °C for 3 min for *co1* and 2 min for *16S* rDNA and *fusA*. For nested PCR of MLST genes, cycling conditions were as follows: first round: 95 °C for 5 min, followed by 15 cycles at 95 °C for 15 s, 55 °C for 30 s, and 72 °C for 40 s; second round: 95 °C for 5 min, followed by 30 cycles at 95 °C for 5 min, 55 °C for 30 s, and 72 °C for 30 s. PCR products were purified with exonuclease I from *E. coli* and sequenced using the BrilliantDye Terminator Cycle Sequencing Kit (Nimagen, Nijmegen, The Netherlands) with the same primers as mentioned for PCR. All sequences were deposited in the GenBank database under accession numbers PX259646-PX259652 for *co1*; PX257974-PX257975 for *Spiroplasma 16S* rDNA gene; PX273486-PX273487 for *Spiroplasma fusA* gene; and PX273488-PX273493, PX273494-PX273499, PX273500-PX273505, PX273506-PX273511, and PX273512-PX273517 for *Wolbachia gatB*, *coxA*, *hcpA*, *ftsZ*, and *fbpA*, respectively (Table S2).

### 2.4. Phylogenetic Analysis

Analysis and assembly of sequences were performed using MEGA12 v.12.0.10 software [60]. All *co1* sequences were checked in the GenBank [https://blast.ncbi.nlm.nih.gov/Blast.cgi (accessed on 1 September 2025)] and BOLD [https://id.boldsystems.org/ (accessed on 1 September 2025)] databases. Sequences of MLST *Wolbachia* loci were checked in the PubMLST [61] database. New *Wolbachia* alleles were additionally checked in the GenBank database. Sequences of *Spiroplasma 16S* rDNA and *fusA* loci were checked in the GenBank database to find the most similar isolates. To reconstruct the phylogenetic relationships of *Wolbachia* isolates, the sequences of MLST genes were concatenated in the order *gatB*-*coxA*-*hcpA*-*ftsZ*-*fbpA* and aligned in MEGA12 using the Muscle algorithm [62]. Additional sequences of the closest isolates were included in the phylogenetic analysis. The total alignment length was 2079 bp. The Kimura two-parameter model of nucleotide substitutions with Gamma distribution (K2G) was chosen as the best fit in MEGA12. For *Spiroplasma 16S* rDNA and *fusA* loci, all sequences, including additional sequences of the closest isolates from the GenBank database, were aligned in MEGA12 using the Muscle algorithm and then trimmed to the shortest sequence. Total alignment lengths were 1011 and 626 bp, respectively. The best-fit model for phylogenetic reconstruction was K2G for *16S* rDNA and General Time Reversible with Gamma distribution for *fusA*. Phylogenetic trees were reconstructed using the Maximum Likelihood (ML) algorithm with 1000 bootstrap iterations.

## 3. Results

Screening for *Wolbachia* and *Spiroplasma* infection revealed seven out of thirty insect stocks that stably harbored symbionts (Table 1). Five stocks were infected with only *Wolbachia*, one stock with only *Spiroplasma*, and the *Nabis* sp. stock harbored both symbionts. Evidence of stable persistence of bacteria was obtained by checking infection status in different insect generations and different insect tissues, and through genetic characterization of each symbiont isolate. In the case of *T. molitor*, we noted that the prevalence of *Spiroplasma* infection varied; not all specimens were infected. Two insect stocks were occasionally characterized by positive signals of *Spiroplasma* infection; however, this result was explained by contamination from the food source. In particular, *Spiroplasma* infection in *A. custos* and *P. maculiventris* stocks could be found only after feeding with *T. molitor* that was truly infected. Moreover, the sequences of *16S* rDNA *Spiroplasma* isolates from *A. custos* and *P. maculiventris* were identical to the bacterial isolate of *T. molitor*. We could not determine whether *Spiroplasma*-positive signals in these two stocks reflected only a trace of symbiont DNA or whether *Spiroplasma* temporarily inhabited these species.

Symbiont-harboring stocks were characterized using the barcoding region of the host mitochondrial *co1* gene (Table 2). Morphological and *co1* barcoding identification of *T. molitor*, *M. pygmaeus,* and *N. ferus* (collection of 2024) were in agreement. However, for *M. pygmaeus*, the complete identity of the barcoding region was also found with *M. caliginosus* in both databases. Two stocks of *Chrysopa* were identified by *co1* barcoding as *C. formosa*, and their sequences differed by two substitutions. The stock of *Nabis* sp. was not identified because several species were closely related by the barcoding sequence. The number of singletons between “*Nabis* sp. (2022)” and “*Nabis ferus* (2024)” was 74 (89% identity). The stock of parasitoid wasp, which was identified by Dr. A. Timokhov as *Trissolcus kozlovi*, showed no similarity with determined species in the BOLD database but had high similarity with *T. kozlovi* in the GenBank database.

**Figure 1:**
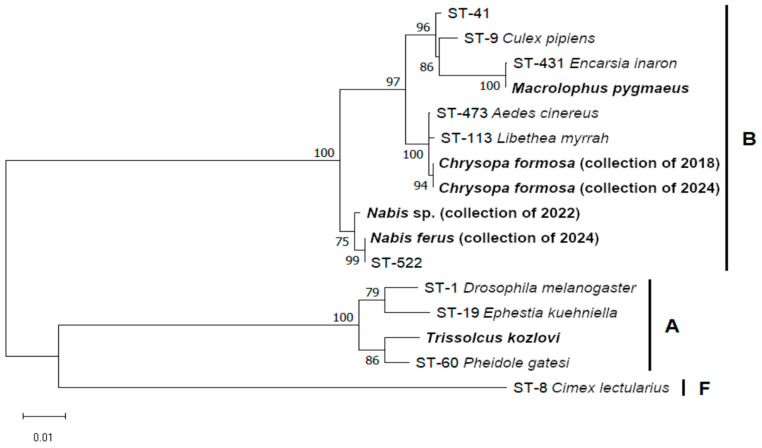
The ML phylogenetic tree of *Wolbachia* isolates based on five concatenated MLST genes (*gatB*-*coxA*-*hcpA*-*ftsZ*-*fbpA*) reconstructed with model K2p + G and 1000 bootstrap iterations. *Wolbachia* sequence types (STs) and/or host species are provided; isolates of this study are in bold. ST-1 and -19, -9 and -41, and -8 were chosen as supergroups A, B, and F references, respectively. Bootstrap values of 75 and higher are indicated.

Five out of six *Wolbachia* isolates belong to supergroup B according to phylogenetic analysis of concatenated MLST genes, and only the isolate of *T. kozlovi* belongs to supergroup A (Figure 1). Only the MLST haplotype of *N. ferus* was previously known (ST-522), while other haplotypes contained new alleles according to both the PubMLST and GenBank databases, as well as new combinations of known alleles. The haplotype of *M. pygmaeus* was very close to ST-431 (one mismatch), which was previously found in the parasitoid wasp *Encarsia inaron* (PubMLST id-1676 and -1677). *Wolbachia* haplotypes of *C. formosa* stocks were identical, and haplotypes of *Nabis* stocks were closely related.

**Figure 2:**
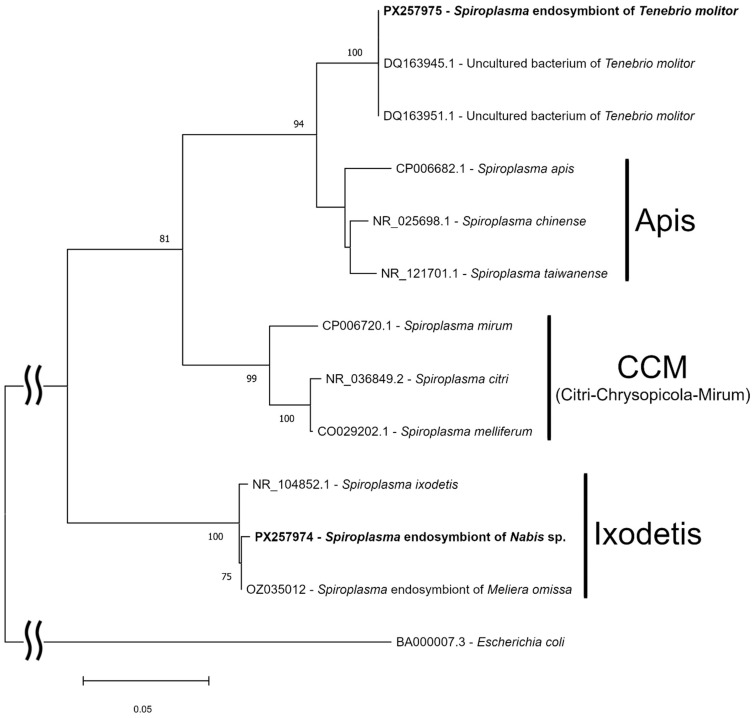
The ML phylogenetic tree of *Spiroplasma* isolates based on *16S* rDNA locus reconstructed with model K2p + G and 1000 bootstrap iterations. GenBank accession numbers and *Spiroplasma* species are provided; isolates of this study are in bold. Bootstrap values of 75 and higher are indicated.

**Figure 3:**
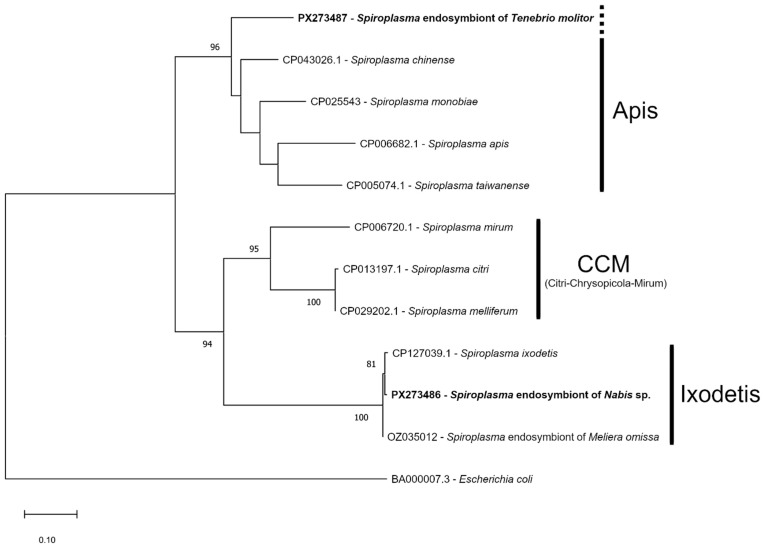
The ML phylogenetic tree of *Spiroplasma* isolates based on *fusA* locus reconstructed with model GTR + G and 1000 bootstrap iterations. GenBank accession numbers and *Spiroplasma* species are provided; isolates of this study are in bold. Bootstrap values of 75 and higher are indicated.

Two isolates of *Spiroplasma* infection were genotyped for *16S* rDNA and *fusA* loci. Reconstruction of phylogenetic relationships revealed that *Spiroplasma* of *Nabis* sp. clustered in the Ixodetis clade, whereas *Spiroplasma* of *T. molitor* was basal to the Apis clade of *Spiroplasma* (Figure 2 and Figure 3). According to the GenBank database, the sequence of *16S* rDNA *Spiroplasma* isolated from *T. molitor* was 99.81% identical to two sequences designated as uncultured bacterium clones (accession numbers DQ163945 and DQ163951), which were also isolated from *T. molitor* [63]. The closest determined *Spiroplasma* was *S. taiwanense* (NR_121701) with 95.72% identity. For the *fusA* gene, the closest isolate was *S. chinense* (CP043026) with 85.27% identity.

## 4. Discussion

### 4.1. Barcoding Identification of the Symbiont-Harbored Species

Since *Wolbachia* and, in some cases, *Spiroplasma* are maternally inherited bacteria, we analyzed the *co1* barcode region for host strains that were found to be infected. In addition to host mtDNA association with symbionts, we were able to compare morphological and genetic identification of the species, and, in most cases, these results were in agreement (Table 2). For *Macrolophus pygmaeus*, there were two species matches: *M. pygmaeus* and *M. caliginosus* (syn. *M. melanotoma*). According to molecular data, they are different species; however, they are hardly distinguishable by morphology [64,65]. The *co1* sequences of *M. caliginosus* that are identical to our data on *M. pygmaeus* were obtained in a study where the authors noted, “*The specimens of M. caliginosus and M. pygmaeus that we received from our sources could not be diagnostically resolved as all samples showed identical sequences*” [66]. Therefore, we assume that our stock is *M. pygmaeus*.

### 4.2. Symbionts in Laboratory Stocks

We found seven laboratory insect stocks that were stably infected with *Spiroplasma* and/or *Wolbachia* bacterial symbionts. Among the studied species, *Spiroplasma* infection was noted in *T. molitor* based on microbiome studies [67,68]; however, there was no particular consideration of *Spiroplasma*. Comparison of the *16S* rDNA sequences of *Spiroplasma* isolates from our and previous reports indicates the same origin of the infection. In the Nabidae family, *Spiroplasma* infection has not been reported previously; thus, our study is the first case of the *Nabis*–*Spiroplasma* symbiotic association.

Regarding *Wolbachia* infection, only *Macrolophus pygmaeus* was previously reported to be infected [69]. Moreover, it was shown that *Wolbachia* induced strong cytoplasmic incompatibility, and it was demonstrated how the infection was distributed in the bug’s organs. In particular, the symbionts were found in salivary glands and ovaries but not in the gut. Other cases of host–*Wolbachia* associations found in our study are new; however, some facts about *Wolbachia* are known in closely related species. For instance, *Wolbachia* infection was reported for *Chrysopa* lacewings [id1163 in the PubMLST database], *Nabis* bugs [70,71], and different *Trissolcus* species [72].

According to previous papers, we could expect to find *Wolbachia* or *Spiroplasma* in some other species studied here. Both symbionts were found in two-spot ladybird (*Adalia bipunctata*) [28,73,74,75,76,77,78] and harlequin ladybird (*Harmonia axyridis*) [75,79,80,81], as well as in the bird cherry-oat aphid (*Rhopalosiphum padi*) [82,83], the black bean aphid (*Aphis fabae*) [20,21,84,85], and in the tomato bug (*Nesidiocoris tenuis*) [86,87]. *Wolbachia* was found in a laboratory stock of house cricket (*Acheta domesticus*) [88] and in the seven-spot ladybird (*Coccinella septempunctata*) [89], the glasshouse whitefly (*Trialeurodes vaporariorum*) [90,91,92,93], the diamondback moth (*Plutella xylostella*) [94,95,96], and the cabbage aphid (*Brevicoryne brassicae*) [97]. *Spiroplasma* was reported in *Zophobas morio* [98] and the white-eyed assassin bug (*Platymeris biguttatus*) [71].

### 4.3. Genetics of the Symbionts

**Figure 4:**
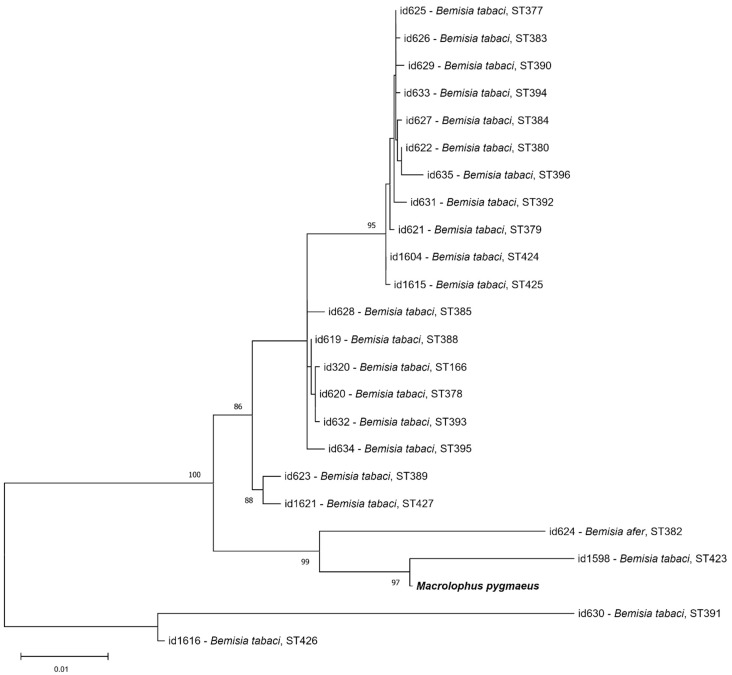
The ML phylogenetic tree of *Wolbachia* haplotypes from *Macrolophus pygmaeus* from our study (in bold) and *Bemisia* species from the PubMLST database based on concatenated MLST sequences. Reconstructed with model T2p + G and 1000 bootstrap iterations. PubMLST id numbers, host species, and *Wolbachia* sequence types (STs) are provided. Bootstrap values of 75 and higher are indicated.

*Wolbachia* variants isolated here belong to supergroups A and B, which are widespread in insects. Only *Wolbachia* MLST haplotype ST-522 found in *N. ferus* (2024) was previously reported in the PubMLST database. Unfortunately, we do not know whether it was found in the same host or not because there are no details on the isolate in the database. Other *Wolbachia* variants in our study contained new alleles or new combination of known alleles. This is the first report of a complete MLST profile of Neuroptera species; two *Wolbachia* isolates of *Chrysopa formosa* have an identical MLST haplotype. Closely related *Wolbachia* variants are found in different insect orders. Previously, a closely related allele of the *fbpA* locus (one substitution) was represented in the PubMLST and GenBank databases (id-1163, KX843371), and also for *Wolbachia* of the *Chrysopa* genus but from Polynesia [99]. This is the first complete MLST profile obtained for a representative of a *Trissolcus* host. Earlier, the diversity of *Wolbachia* in five *Trissolcus* species was characterized by the *wsp* gene [72]. The authors observed similar variants that clustered into supergroup B. In contrast, the isolate of *T. kozlovi* belonged to supergroup A of *Wolbachia*. The haplotype of *M. pygmaeus* contained previously known alleles but in a new combination. The most similar haplotype was found in parasitoid wasp *Encarsia inaron*, which is used in biocontrol against whiteflies. *E. inaron* and *M. pygmaeus* could share the same whiteflies as food substrate, and therefore this could be an arena of *Wolbachia* exchange between predator species. Such a pattern of *Wolbachia* horizontal transmission was presented by Ahmed et al. [11]. In the case of *M. pygmaeus*, the symbiont *Wolbachia* was present in salivary glands [69], and feeding by this predator occurred by injecting saliva into prey and sucking out digestive products. This process can be interrupted at the saliva injection stage, and the prey stays alive but has *Wolbachia* bacteria from *Macrolophus*. Also, the predator could hypothetically acquire new *Wolbachia* from prey at the sucking out stage. Of course, such speculation should be tested in experiments. Here, we note that Machtelinckx et al. [69] indicated that “Performing MLST may provide more evidence on the phylogenetic relationship between the *Wolbachia* strains in the whitefly and the mirid bug”. We compared *Wolbachia* haplotypes of *Bemisia tabaci* from the PubMLST database [https://pubmlst.org/bigsdb?db=pubmlst_wolbachia_isolates (accessed on 1 September 2025)] with our isolate of *M. pygmaeus* and found no similarities (Figure 4). However, it is obvious that the genetic diversity of *Wolbachia* in mirid bugs and whiteflies was underestimated. For instance, a number of *Wolbachia* variants were reported only for one species, *Bemisia tabaci* (Figure 4). *M. pygmaeus* also had different *Wolbachia* variants. Machtelinckx et al. [69] sequenced one out of five MLST loci (*fbpA*), and it drastically differed from the allele found in our isolate. Therefore, the finding of the same variants in prey and predator cannot be ruled out; however, direct evidence of such symbiont transmission should be obtained in an experiment.

Two *Spiroplasma* isolates found here are distantly related according to conserved genetic markers. *Spiroplasma* of *Nabis* sp. belongs to the Ixodetis clade, the most widespread in arthropods [26], while the isolate of *T. molitor* is a unique branch that is basal to the Apis clade. The sequences of our and previously reported isolates of *T. molitor* are identical (Figure 2), which indicates broad distribution of this infection in *T. molitor* stocks. It is known that spiroplasmas of the Ixodetis clade can have different effects on hosts [2]. They can increase hatchability and decrease development time [47] and defend against parasitoids and fungal pathogens [100,101], but can also induce male killing [30,33,102,103,104] and, moreover, can be pathogenic for hosts [105]. Therefore, some of these effects can be expected in *Nabis* sp. There are no data on any *Spiroplasma* effects on *T. molitor* biology, and the unique placement of the isolate found in our study only increases interest in studying this symbiotic association.

We should note that there are two cautions in screening for *Wolbachia* and *Spiroplasma*, especially in non-model species. First is the risk of obtaining false-positive signals due to transient microbiota from infected substrates and the frequent presence of “weed” mites (e.g., *Tyrophagous* spp.) in insect stocks. Second is the risk of obtaining false-negative signals due to variation in infection prevalence in insect stocks, which could be a result of the imperfect transmission of infection [33,106] and low bacterial density in individuals or in some organs and tissues [107,108].

### 4.4. How These Symbionts Can Be Used in Biocontrol

In the present study, we firstly reported on *Wolbachia* and *Spiroplasma* in species with potential for biocontrol. Specifically, *Wolbachia* and *Spiroplasma* were detected in the predatory bug *Nabis ferus*. Additionally, *Wolbachia* was identified for the first time in *Chrysopa formosa* and *Trissolcus kozlovi*. All but one of the *Wolbachia* isolates were characterized by novel MLST alleles or novel combinations of previously known alleles. Also, we provide the first genetic characterization of *Spiroplasma* isolated from *Tenebrio molitor*. These findings pave the way for further investigation into the roles of these symbionts in their hosts’ biology.

The application of *Wolbachia* and *Spiroplasma* in agriculture is at the initial stages; there are as yet no use-ready technologies for these symbionts in applied science. However, several promising directions for their utilization can be identified. Notably, *Wolbachia* strains from supergroup B found in *Trichogramma* wasps, widely used agents in biological pest control, are known to induce parthenogenesis, resulting in the production of all-female offspring (thelytoky) [109]. By transferring the thelytokous *Wolbachia* strains to bisexual *Trichogramma* stocks, it is possible to create female-only lines, which can significantly lower the cost of mass wasp production and enhance the efficiency of biocontrol programs [110]. Furthermore, certain *Wolbachia* strains possess genes conferring nutritional independence, such as the biotin operon [111]. The introduction of such strains into biocontrol species could improve the sustainability and management of production stocks. The defensive role of *Spiroplasma* [2] could be used for applied species to protect them from natural enemies [112,113,114] or even from abiotic factors [115]. In summary, while technologies utilizing *Wolbachia* and *Spiroplasma* are not yet available, active research supports their potential for improving efficiency, cost-effectiveness, and environmental resilience in agriculture.

## 5. Conclusions

Some of the symbiont-harboring species considered here are maintained in laboratory stocks worldwide, which means that other researchers can find *Wolbachia* and *Spiroplasma* in their own stocks. Our other original stocks that also harbor symbionts can be successfully established and maintained elsewhere because they are easily cultivated under laboratory conditions. We also list species that are cultivated and could be infected according to previous reports. All of them are potential sources for fundamental studies of symbiont–host interactions, as well as for applied research to improve productivity, cultivation methods, and other aspects of practical insect use.

## Figures and Tables

**Table 1:** List of species maintained in the Laboratory and their characteristics: purpose of use, origin, nutrient substrate, and infection status.

Order	Species	Purpose of Culture	Origin (Year)	Nutrient Substrate	*Wolbachia*	*Spiroplasma*
Orthoptera	*Acheta domesticus*	IF	PS, Msk, (2022)	Vegetables	0	0
Coleoptera	*Adalia bipunctata*	BC	SFSCA, Nsk (2024)	Aphids	0	0
Hemiptera	*Aphis fabae*	test, IF	SFSCA, Nsk (2017)	Bean	0	0
Hemiptera	*Arma custos*	BC	SFSCA, Nsk (2023)	*Tenebrio molitor*	0	0*
Hemiptera	*Brevicoryne brassicae*	test, IF	SFSCA, Nsk (2023)	Cabbage	0	0
Coleoptera	*Bruchus rufimanus*	test	PS, Msk (2024)	Mash	0	0
Neuroptera	*Chrysopa formosa* 1	BC	SFSCA, Nsk (2018)	Aphids	1	0
Neuroptera	*Chrysopa formosa* 2	BC	SFSCA, Nsk, (2024)	Aphids	1	0
Coleoptera	*Coccinella septempunctata*	BC	SFSCA, Nsk (2022)	Aphids	0	0
Orthoptera	*Grillus bimaculatus*	IF	PS, Msk (2022)	Vegetables	0	0
Coleoptera	*Harmonia axyridis*	BC	SFSCA, Nsk (2022)	Aphids	0	0
Coleoptera	*Harmonia (Leis) dimidiata*	BC	VIZR, SPb (2024)	Aphids	0	0
Coleoptera	*Hippodamia variegata*	BC	SFSCA, Nsk (2022)	Aphids	0	0
Lepidoptera	*Lacanobia oleracea*	test, IF	SFSCA, Nsk (2023)	Cabbage	0	0
Hemiptera	*Macrolophus pygmaeus*	BC	CS (2017)	*T. vaporariorum*	1	0
Hemiptera	*Megoura viciae*	test, IF	SFSCA, Nsk (2017)	Bean	0	0
Hemiptera	*Nabis* sp.	BC	SFSCA, Nsk (2023)	Aphids	1	1
Hemiptera	*Nabis ferus*	BC	SFSCA, Nsk (2024)	Aphids	1	0
Hemiptera	*Nesidiocoris tenuis*	BC	VIZR, SPb (2023)	*T. vaporariorum*	0	0
Hemiptera	*Platymeris biguttatus*	test	PS, T (2023)	*T. molitor, Z. morio*	0	0
Lepidoptera	*Plutella xylostella*	IF	SFSCA, Nsk (2019)	Rape	0	0
Hemiptera	*Podisus maculiventris*	BC	VIZR (2017)	*T. molitor, Z. morio*	0	0*
Hemiptera	*Psyttala horrida*	test	PS, K (2024)	*T. molitor, Z. morio*	0	0
Hemiptera	*Rhopalosiphum padi*	test, IF	SFSCA, Nsk (2018)	Wheat	0	0
Hemiptera	*Schizaphis graminum*	test, IF	SFSCA, Nsk (2017)	Wheat	0	0
Coleoptera	*Subcoccinella vigintiquatuorpunctata*	test	SFSCA, Nsk (2023)	Alfalfa	0	0
Coleoptera	*Tenebrio molitor*	test, IF	PS, Nsk (2020)	Bran, vegetables	0	1
Hemiptera	*Trialeurodes vaporariorum*	test, IF	SFSCA, Nsk (2021)	Tobacco	0	0
Hymenoptera	*Trissolcus kozlovi*	BC	SFSCA, Nsk (2021)	*P. maculiventris*	1	0
Coleoptera	*Zophobas morio*	IF	PS, Nsk (2020)	Bran, vegetables	0	0

Footnotes: IF—insect food, BC—biological control, test—for experimental procedures, SFSCA—Siberian Federal Research Centre of Agro-BioTechnologies of the Russian Academy of Sciences, VIZR—All-Russian Institute of Plant Protection, PS—private stock, CS—commercial stock, SPb—Saint Petersburg, Nsk—Novosibirsk Province, Msk—Moscow city, K—Kaluga city, T—Tolyatti, 0*—uninfected but occasionally false-positive signal due to contamination by a food substrate.

**Table 2:** Morphological and barcoding identification of *Spiroplasma*-/*Wolbachia*-infected species.

Morphological Identification (GenBank Acc. Num.)	BOLD (% Identity)	GenBank (% Identity)
*Trissolcus kozlovi* (PX259647)	Scelionidae (99.53)	*T. kozlovi* (99.12)
*Tenebrio molitor* (PX259651)	*T. molitor* (99.85)	*T. molitor* (99.85)
*Nabis* sp. (PX259649)	*N. rugosus*, *N. pseudoferus*, *N. brevis* (99.8), *N. ericetorum* (99.7), *N. ferus* (99.54)	*N. rugosus*, *N. pseudoferus* (99.7), *N. ferus* (99.54)
*Nabis ferus* (PX259650)	*N. ferus* (99.7)	*N. ferus* (99.54)
*Chrysopa formosa* 1 (PX259648)	*C. formosa* (100)	*C. formosa* (100)
*Chrysopa formosa* 2 (PX259652)	*C. formosa* (99.85)	*C. formosa* (99.7)
*Macrolophus pygmaeus* (PX259646)	*M. pygmaeus*, *M. caliginosus* (100)	*M. pygmaeus*, *M. caliginosus* (100)

## Data Availability

The original contributions presented in this study are included in the article/Supplementary Materials. Further inquiries can be directed to the corresponding author.

## Supplementary Materials

The following supporting information can be downloaded at https://www.mdpi.com/article/10.3390/insects16111168/s1. Table S1: Life stages and DNA extraction sources; Table S2: Number of specimens examined and sequenced for each stock.

## References

[1] Werren J.H. (1997). Biology of *Wolbachia*. Annu. Rev. Entomol..

[2] Ballinger M.J., Perlman S.J. (2019). The defensive *Spiroplasma*. Curr. Opin. Insect Sci..

[3] Shishkina O.D., Gruntenko N.E. (2025). Symbiosis of intracellular bacteria *Wolbachia* with insects: A hundred years of study summarized. Vavilov J. Genet. Breed..

[4] Hilgenboecker K., Hammerstein P., Schlattmann P., Telschow A., Werren J.H. (2008). How many species are infected with *Wolbachia*?—A statistical analysis of current data. FEMS Microbiol. Lett..

[5] Zug R., Hammerstein P. (2012). Still a host of hosts for *Wolbachia*: Analysis of recent data suggests that 40% of terrestrial arthropod species are infected. PLoS ONE.

[6] Sazama E.J., Bosch M.J., Shouldis C.S., Ouellette S.P., Wesner J.S. (2017). Incidence of *Wolbachia* in aquatic insects. Ecol. Evol..

[7] Fenn K., Blaxter M. (2006). *Wolbachia* genomes: Revealing the biology of parasitism and mutualism. Trends Parasitol..

[8] Haegeman A., Vanholme B., Jacob J., Vandekerckhove T.T., Claeys M., Borgonie G., Gheysen G. (2009). An endosymbiotic bacterium in a plant-parasitic nematode: Member of a new *Wolbachia* supergroup. Int. J. Parasitol..

[9] Kittayapong P., Jamnongluk W., Thipaksorn A., Milne J.R., Sindhusake C. (2003). *Wolbachia* infection complexity among insects in the tropical rice-field community. Mol. Ecol..

[10] Stahlhut J.K., Desjardins C.A., Clark M.E., Baldo L., Russell J.A., Werren J.H., Jaenike J. (2010). The mushroom habitat as an ecological arena for global exchange of *Wolbachia*. Mol. Ecol..

[11] Ahmed M.Z., Li S.J., Xue X., Yin X.J., Ren S.X., Jiggins F.M., Greeff J.M., Qiu B.L. (2015). The intracellular bacterium *Wolbachia* uses parasitoid wasps as phoretic vectors for efficient horizontal transmission. PLoS Pathog..

[12] Werren J.H., Windsor D., Guo L.R. (1995). Distribution of *Wolbachia* among neotropical arthropods. Proc. R. Soc. Lond. Ser. B Biol. Sci..

[13] Bandi C., Anderson T.J., Genchi C., Blaxter M.L. (1998). Phylogeny of *Wolbachia* in filarial nematodes. Proc. R. Soc. Lond. Ser. B Biol. Sci..

[14] Lo N., Casiraghi M., Salati E., Bazzocchi C., Bandi C. (2002). How many *Wolbachia* supergroups exist?. Mol. Biol. Evol..

[15] Bordenstein S., Rosengaus R.B. (2005). Discovery of a novel *Wolbachia* supergroup in Isoptera. Curr. Microbiol..

[16] Ros V.I., Fleming V.M., Feil E.J., Breeuwer J.A. (2009). How diverse is the genus *Wolbachia*? Multiple-gene sequencing reveals a putatively new *Wolbachia* supergroup recovered from spider mites (Acari: Tetranychidae). Appl. Environ. Microbiol..

[17] Augustinos A.A., Santos-Garcia D., Dionyssopoulou E., Moreira M., Papapanagiotou A., Scarvelakis M., Doudoumis V., Ramos S., Aguiar A.F., Borges P.A.V. (2011). Detection and characterization of *Wolbachia* infections in natural populations of aphids: Is the hidden diversity fully unraveled?. PLoS ONE.

[18] Wang Z., Su X.M., Wen J., Jiang L.Y., Qiao G.X. (2014). Widespread infection and diverse infection patterns of *Wolbachia* in Chinese aphids. Insect Sci..

[19] Glowska E., Dragun-Damian A., Dabert M., Gerth M. (2015). New *Wolbachia* supergroups detected in quill mites (Acari: Syringophilidae). Infect. Genet. Evol..

[20] Lefoulon E., Clark T., Borveto F., Perriat-Sanguinet M., Moulia C., Slatko B.E., Gavotte L. (2020). Pseudoscorpion *Wolbachia* symbionts: Diversity and evidence for a new supergroup S. BMC Microbiol..

[21] Sharma A.K., Som A. (2023). Assigning new supergroups V and W to the *Wolbachia* diversity. Bioinformation.

[22] Duron O., Bouchon D., Boutin S., Bellamy L., Zhou L., Engelstädter J., Hurst G.D. (2008). The diversity of reproductive parasites among arthropods: *Wolbachia* do not walk alone. BMC Biol..

[23] Cisak E., Wójcik-Fatla A., Zajac V., Sawczyn A., Sroka J., Dutkiewicz J. (2015). *Spiroplasma*—An emerging arthropod-borne pathogen?. Ann. Agric. Environ. Med..

[24] Gasparich G.E., Whitcomb R.F., Dodge D., French F.E., Glass J., Williamson D.L. (2004). The genus *Spiroplasma* and its non-helical descendants: Phylogenetic classification, correlation with phenotype and roots of the Mycoplasma mycoides clade. Int. J. Syst. Evol. Microbiol..

[25] Bolanos L.M., Servin-Garciduenas L.E., Martinez-Romero E. (2015). Arthropod–*Spiroplasma* relationship in the genomic era. FEMS Microbiol. Ecol..

[26] Binetruy F., Bailly X., Chevillon C., Martin O.Y., Bernasconi M.V., Duron O. (2019). Phylogenetics of the *Spiroplasma ixodetis* endosymbiont reveals past transfers between ticks and other arthropods. Ticks Tick-Borne Dis..

[27] Poulson D.F., Sakaguchi B. (1961). Nature of “sex-ratio” agent in *Drosophila*. Science.

[28] Hurst G.D.D., von der Schulenburg J.G., Majerus T.M.O., Bertrand D., Zakharov I.A., Baungaard J., Völkl W., Stouthamer R., Majerus M.E.N. (1999). Invasion of one insect species, *Adalia bipunctata*, by two different male-killing bacteria. Insect Mol. Biol..

[29] Williamson D.L., Sakaguchi B., Hackett K.J., Whitcomb R.F., Tully J.G., Carle P., Bové J.M., Adams J.R., Konai M., Henegar R.B. (1999). *Spiroplasma poulsonii* sp. nov., a new species associated with male-lethality in *Drosophila willistoni*, a neotropical species of fruit fly. Int. J. Syst. Evol. Microbiol..

[30] Tinsley M.C., Majerus M.E.N. (2006). A new male-killing parasitism: *Spiroplasma* bacteria infect the ladybird beetle *Anisosticta novemdecimpunctata* (Coleoptera: Coccinellidae). Parasitology.

[31] Jiggins, Hurst, Dolman, Majerus (2000). High-prevalence male-killing *Wolbachia* in the butterfly *Acraea encedana*. J. Evol. Biol..

[32] Werren J.H., Baldo L., Clark M.E. (2008). *Wolbachia*: Master manipulators of invertebrate biology. Nat. Rev. Microbiol..

[33] Tabata J., Hattori Y., Sakamoto H., Yukuhiro F., Fujii T., Kugimiya S., Mochizuki A., Ishikawa Y., Kageyama D. (2011). Male killing and incomplete inheritance of a novel *Spiroplasma* in the moth *Ostrinia zaguliaevi*. Microb. Ecol..

[34] Pollmann M., Moore L.D., Krimmer E., D’Alvise P., Hasselmann M., Perlman S.J., Ballinger M.J., Steidle J.L.M., Gottlieb Y. (2022). Highly transmissible cytoplasmic incompatibility by the extracellular insect symbiont *Spiroplasma*. iScience.

[35] Starr D.J., Cline T.W. (2002). A host–parasite interaction rescues *Drosophila* oogenesis defects. Nature.

[36] Brownlie J.C., Cass B.N., Riegler M., Witsenburg J.J., Iturbe-Ormaetxe I., McGraw E.A., O’Neill S.L. (2009). Evidence for metabolic provisioning by a common invertebrate endosymbiont, *Wolbachia pipientis*, during periods of nutritional stress. PLoS Pathog..

[37] Ikeya T., Broughton S., Alic N., Grandison R., Partridge L. (2009). The endosymbiont *Wolbachia* increases insulin/IGF-like signalling in *Drosophila*. Proc. R. Soc. Lond. Ser. B Biol. Sci..

[38] Zug R., Hammerstein P. (2015). Bad guys turned nice? A critical assessment of *Wolbachia* mutualisms in arthropod hosts. Biol. Rev..

[39] Jaenike J., Stahlhut J.K., Boelio L.M., Unckless R.L. (2010). Association between *Wolbachia* and *Spiroplasma* within *Drosophila neotestacea*: An emerging symbiotic mutualism?. Mol. Ecol..

[40] Xie J., Vilchez I., Mateos M. (2010). *Spiroplasma* bacteria enhance survival of *Drosophila hydei* attacked by the parasitic wasp *Leptopilina heterotoma*. PLoS ONE.

[41] Higashi C.H., Patel V., Kamalaker B., Inaganti R., Bressan A., Russell J.A., Oliver K.M. (2024). Another tool in the toolbox: Aphid-specific *Wolbachia* protect against fungal pathogens. Environ. Microbiol..

[42] Perlmutter J.I., Atadurdyyeva A., Schedl M.E., Unckless R.L. (2025). *Wolbachia* enhances the survival of *Drosophila* infected with fungal pathogens. BMC Biol..

[43] Mouches C., Bové J.M., Albisetti J. (1984). Pathogenicity of *Spiroplasma apis* and other spiroplasmas for honey-bees in Southwestern France. Ann. L’Institut Pasteur/Microbiol..

[44] Lorenz B., Schroeder J., Reischl U. (2002). First evidence of an endogenous *Spiroplasma* sp. infection in humans manifesting as unilateral cataract associated with anterior uveitis in a premature baby. Graefes Arch. Clin. Exp. Ophthalmol..

[45] Santa Olga Cacciola A.B., Pane A., Furneri P.M., Gill H., Garg H. (2017). *Spiroplasma* spp.: A plant, arthropod, animal and human pathogen. Citrus Pathology.

[46] Zhang Y.K., Yang K., Zhu Y.X., Hong X.Y. (2018). Symbiont-conferred reproduction and fitness benefits can favour their host occurrence. Ecol. Evol..

[47] Xie K., Lu Y.J., Yang K., Huo S.M., Hong X.Y. (2020). Co-infection of *Wolbachia* and *Spiroplasma* in spider mite *Tetranychus truncatus* increases male fitness. Insect Sci..

[48] Zhu Y.X., Song Z.R., Song Y.L., Hong X.Y. (2020). Double infection of *Wolbachia* and *Spiroplasma* alters induced plant defense and spider mite fecundity. Pest Manag. Sci..

[49] Tarbinsky S.P., Plavilshchikov N.N. (1948). Opredelitel Nasekomykh Evropeiskoi Chasti SSSR [Key to the Insects of the European Part of the USSR].

[50] Bei-Bienko G.Y. (1965). Zhestkokrylye i verokrylye [Coleoptera and Strepsiptera]. Opredelitel Nasekomykh Evropeiskoi Chasti SSSR [Key to the Insects of the European Part of the USSR].

[51] Leley A.S. (1988). Opredelitel Nasekomykh Dalnego Vostoka SSSR. Tom II. Ravnokrylye i Poluzhestkokrylye [Key to the Insects of the Soviet Far East. Vol. II. Homoptera and Heteroptera].

[52] Vinokurov N.N., Kanyukova E.V. (1995). Heteropteran Insects (Heteroptera) of Siberia.

[53] Andreeva I.V., Shatalova E.I., Ulyanova E.G. (2020). Sposob Razvedeniya Kapustnoi Moli Plutella xylostella L. [A Method for Breeding the Diamondback Moth Plutella xylostella L.]. Russian Patent.

[54] Andreeva I.V., Khodakova A.V., Shatalova E.I. (2025). Sposob Razvedeniya Laboratornoi Kultury Entomofaga Trissolcus kozlovi Rjachovsky [A Method for Breeding a Laboratory Culture of the Entomophagous Trissolcus kozlovi Rjachovsky]. Russian Patent.

[55] Shatalova E.I., Agricolyanskaya N.I., Andreeva I.V., Ulyanova E.G., Khodakova A.V. (2023). Sposob Razvedeniya Klopa Podizusa Podisus maculiventris Say [A Method for Breeding the Spined Soldier Bug Podisus maculiventris Say]. Russian Patent.

[56] Vrijenhoek R. (1994). DNA primers for amplification of mitochondrial cytochrome c oxidase subunit I from diverse metazoan invertebrates. Mol. Mar. Biol. Biotechnol..

[57] Baldo L., Dunning Hotopp J.C., Jolley K.A., Bordenstein S.R., Biber S.A., Choudhury R.R., Hayashi C., Maiden M.C.J., Tettelin H., Werren J.H. (2006). Multilocus sequence typing system for the endosymbiont *Wolbachia pipientis*. Appl. Environ. Microbiol..

[58] Ilinsky Y. (2013). Coevolution of *Drosophila melanogaster* mtDNA and *Wolbachia* genotypes. PLoS ONE.

[59] Sanada-Morimura S., Matsumura M., Noda H. (2013). Male killing caused by a *Spiroplasma* symbiont in the small brown planthopper, *Laodelphax striatellus*. J. Hered..

[60] Kumar S., Stecher G., Suleski M., Sanderford M., Sharma S., Tamura K. (2024). MEGA12: Molecular Evolutionary Genetic Analysis version 12 for adaptive and green computing. Mol. Biol. Evol..

[61] Jolley K.A., Bray J.E., Maiden M.C. (2018). Open-access bacterial population genomics: BIGSdb software, the PubMLST.org website and their applications. Wellcome Open Res..

[62] Edgar R.C. (2004). MUSCLE: Multiple sequence alignment with high accuracy and high throughput. Nucleic Acids Res..

[63] Dunn A.K., Stabb E.V. (2005). Culture-independent characterization of the microbiota of the ant lion *Myrmeleon mobilis* (Neuroptera: Myrmeleontidae). Appl. Environ. Microbiol..

[64] Martinez-Cascales J.I., Cenis J.L., Sanchez J.A. (2006). Differentiation of *Macrolophus pygmaeus* (Rambur 1839) and *Macrolophus melanotoma* (Costa 1853) (Heteroptera: Miridae) based on molecular data. Bull. OILB/SROP.

[65] Castañé C., Agustí N., Arnó J., Gabarra R., Riudavets J., Comas J., Alomar O. (2013). Taxonomic identification of *Macrolophus pygmaeus* and *Macrolophus melanotoma* based on morphometry and molecular markers. Bull. Entomol. Res..

[66] Pasquer F., Pfunder M., Frey B., Frey J.E. (2009). Microarray-based genetic identification of beneficial organisms as a new tool for quality control of laboratory cultures. Biocontrol. Sci. Technol..

[67] Jung J., Heo A., Park Y.W., Kim Y.J., Koh H., Park W. (2014). Gut microbiota of *Tenebrio molitor* and their response to environmental change. J. Microbiol. Biotechnol..

[68] Wang Y., Zhang Y. (2015). Investigation of gut-associated bacteria in *Tenebrio molitor* (Coleoptera: Tenebrionidae) larvae using culture-dependent and DGGE methods. Ann. Entomol. Soc. Am..

[69] Machtelinckx T., Van Leeuwen T., Vanholme B., Gehesquière B., Dermauw W., Vandekerkhove B., Gheysen G., De Clercq P. (2009). *Wolbachia* induces strong cytoplasmic incompatibility in the predatory bug *Macrolophus pygmaeus*. Insect Mol. Biol..

[70] Kikuchi Y., Fukatsu T. (2003). Diversity of *Wolbachia* endosymbionts in heteropteran bugs. Appl. Environ. Microbiol..

[71] Li G., Sun J., Meng Y., Yang C., Chen Z., Wu Y., Tian L., Song F., Cai W., Zhang X. (2022). The impact of environmental habitats and diets on the gut microbiota diversity of true bugs (Hemiptera: Heteroptera). Biology.

[72] Guz N., Kocak E., Emre Akpinar A., Oktay Gurkan M., Neset Kilincer A. (2012). *Wolbachia* infection in *Trissolcus* species (Hymenoptera: Scelionidae). Eur. J. Entomol..

[73] Zakharov I.A., Goriacheva I.I., Shaĭkevich E.V., Schulenburg J.H., Majerus E.N. (2000). *Wolbachia*—A new bacteria causing sex ratio bias in the two-spot lady-bird *Adalia bipunctata* L.. Genetika.

[74] Sokolova M.I., Zinkevich N.S., Zakharov I.A. (2002). Bacteria in ovarioles of females from maleless families of ladybird beetles *Adalia bipunctata* L. (Coleoptera: Coccinellidae) naturally infected with *Rickettsia*, *Wolbachia*, and *Spiroplasma*. J. Invertebr. Pathol..

[75] Ahmed M.Z., Ren S.X., Mandour N.S., Greeff J.M., Qiu B.L. (2010). Prevalence of *Wolbachia* supergroups A and B in *Bemisia tabaci* (Hemiptera: Aleyrodidae) and some of its natural enemies. J. Econ. Entomol..

[76] Shaikevich E.V., Romanov D.A., Zakharov I.A. (2021). The diversity of *Wolbachia* and its effects on host reproduction in a single *Adalia bipunctata* (Coleoptera: Coccinellidae) population. Symbiosis.

[77] Romanov D.A., Zakharov I.A. (2023). Distribution of symbiotic bacteria *Spiroplasma*, *Rickettsia*, *Wolbachia* in populations of *Adalia bipunctata* (Linnaeus, 1758). Symbiosis.

[78] Archer J., Hurst G.D., Hornett E.A. (2023). Male-killer symbiont screening reveals novel associations in *Adalia* ladybirds. Access Microbiol..

[79] Goryacheva I.I., Blekhman A.V., Andrianov B.V., Gorelova T.V., Zakharov I.A. (2015). Genotypic diversity of *Wolbachia* pipientis in native and invasive *Harmonia axyridis* Pall., 1773 (Coleoptera, Coccinellidae) populations. Russ. J. Genet..

[80] Goryacheva I., Blekhman A., Andrianov B., Romanov D., Zakharov I. (2018). *Spiroplasma* infection in *Harmonia axyridis*-Diversity and multiple infection. PLoS ONE.

[81] Awad M., Piálková R., Haelewaters D., Nedvěd O. (2023). Infection patterns of *Harmonia axyridis* (Coleoptera: Coccinellidae) by ectoparasitic microfungi and endosymbiotic bacteria. J. Invertebr. Pathol..

[82] Guo J., Liu X., Poncelet N., He K., Francis F., Wang Z. (2019). Detection and geographic distribution of seven facultative endosymbionts in two *Rhopalosiphum* aphid species. MicrobiologyOpen.

[83] Majeed M.Z., Sayed S., Bo Z., Raza A., Ma C.S. (2022). Bacterial symbionts confer thermal tolerance to cereal aphids *Rhopalosiphum padi* and *Sitobion avenae*. Insects.

[84] Zytynska S.E., Meyer S.T., Sturm S., Ullmann W., Mehrparvar M., Weisser W.W. (2016). Secondary bacterial symbiont community in aphids responds to plant diversity. Oecologia.

[85] Romanov D.A., Zakharov I.A., Shaikevich E.V. (2020). *Wolbachia*, *Spiroplasma*, and *Rickettsia* symbiotic bacteria in aphids (Aphidoidea). Vavilov J. Genet. Breed..

[86] Owashi Y., Minami T., Kikuchi T., Yoshida A., Nakano R., Kageyama D., Adachi-Hagimori T. (2023). Microbiome of zoophytophagous biological control agent *Nesidiocoris tenuis*. Microb. Ecol..

[87] Shibata T., Shimoda M., Kobayashi T., Arai H., Owashi Y., Uehara T. (2024). High-quality genome of the zoophytophagous stink bug, *Nesidiocoris tenuis*, informs their food habit adaptation. G3.

[88] Fernandez-Cassi X., Söderqvist K., Bakeeva A., Vaga M., Dicksved J., Vagsholm I., Jansson A., Boqvist S. (2020). Microbial communities and food safety aspects of crickets (*Acheta domesticus*) reared under controlled conditions. J. Insects Food Feed.

[89] Weinert L.A., Tinsley M.C., Temperley M., Jiggins F.M. (2007). Are we underestimating the diversity and incidence of insect bacterial symbionts? A case study in ladybird beetles. Biol. Lett..

[90] Škaljac M., Žanić K., Hrnčić S., Radonjić S., Perović T., Ghanim M. (2013). Diversity and localization of bacterial symbionts in three whitefly species (Hemiptera: Aleyrodidae) from the east coast of the Adriatic Sea. Bull. Entomol. Res..

[91] Skaljac M., Kanakala S., Zanic K., Puizina J., Lepen Pleic I., Ghanim M. (2017). Diversity and phylogenetic analyses of bacterial symbionts in three whitefly species from Southeast Europe. Insects.

[92] Kapantaidaki D.E., Ovčarenko I., Fytrou N., Knott K.E., Bourtzis K., Tsagkarakou A. (2015). Low levels of mitochondrial DNA and symbiont diversity in the worldwide agricultural pest, the greenhouse whitefly *Trialeurodes vaporariorum* (Hemiptera: Aleyrodidae). J. Hered..

[93] Tat Z., Koçak E. (2024). Composition of Secondary Endosymbiont Bacteria in Two Whitefly Species (Hemiptera: Aleyrodidae). Akdeniz Univ. Ziraat Fak. Derg..

[94] Jeyaprakash A., Hoy M.A. (2000). Long PCR improves *Wolbachia* DNA amplification: *Wsp* sequences found in 76% of sixty-three arthropod species. Insect Mol. Biol..

[95] Delgado A.M., Cook J.M. (2009). Effects of a sex-ratio distorting endosymbiont on mtDNA variation in a global insect pest. BMC Evol. Biol..

[96] Zhu X., Liu T., He A., Zhang L., Li J., Li T., Miao X., You M., You S. (2023). Diversity of *Wolbachia* infection and its influence on mitochondrial DNA variation in the diamondback moth, *Plutella xylostella*. Mol. Phylogenet. Evol..

[97] Jiang X.F., Wang L., Zhang L., Luo L.Z. (2009). Molecular detection of *Wolbachia* in three species of vegetable aphids collected from Beijing suburb. Plant Prot..

[98] Eddoubaji Y., Aldeia C., Campos-Madueno E.I., Moser A.I., Kundlacz C., Perreten V., Hilty M., Endimiani A. (2024). A new in vivo model of intestinal colonization using *Zophobas morio* larvae: Testing hyperepidemic ESBL-and carbapenemase-producing Escherichia coli clones. Front. Microbiol..

[99] Bailly-Bechet M., Martins-Simões P., Szöllősi G.J., Mialdea G., Sagot M.F., Charlat S. (2017). How long does *Wolbachia* remain on board?. Mol. Biol. Evol..

[100] Łukasik P., van Asch M., Guo H., Ferrari J., Charles J., Godfray H. (2013). Unrelated facultative endosymbionts protect aphids against a fungal pathogen. Ecol. Lett..

[101] McLean A.H.C., Hrček J., Parker B.J., Mathé-Hubert H., Kaech H., Paine C., Godfray H.C.J. (2020). Multiple phenotypes conferred by a single insect symbiont are independent. Proc. R. Soc. Lond. Ser. B Biol. Sci..

[102] Majerus T.M.O., von der Schulenburg J.H., Majerus M.E.N., Hurst G.D.D. (1999). Molecular identification of a male-killing agent in the ladybird *Harmonia axyridis* (Pallas) (Coleoptera: Coccinellidae). Insect Mol. Biol..

[103] Majerus M.E., Hinrich J., Schulenburg G.V.D., Zakharov I.A. (2000). Multiple causes of male-killing in a single sample of the two-spot ladybird, *Adalia bipunctata* (Coleoptera: Coccinellidae) from Moscow. Heredity.

[104] Takamatsu T., Arai H., Abe N., Nakai M., Kunimi Y., Inoue M.N. (2021). Coexistence of two male-killers and their impact on the development of oriental tea tortrix *Homona magnanima*. Microb. Ecol..

[105] Nai Y.S., Su P.Y., Hsu Y.H., Chiang C.H., Kim J.S., Chen Y.W., Wang C.H. (2014). A new spiroplasma isolate from the field cricket (Gryllus bimaculatus) in Taiwan. J. Invertebr. Pathol..

[106] Hoffmann A.A., Hercus M., Dagher H. (1998). Population dynamics of the *Wolbachia* infection causing cytoplasmic incompatibility in *Drosophila melanogaster*. Genetics.

[107] Arthofer W., Riegler M., Avtzis D.N., Stauffer C. (2009). Evidence for low-titre infections in insect symbiosis: *Wolbachia* in the bark beetle *Pityogenes chalcographus* (Coleoptera, Scolytinae). Environ. Microbiol..

[108] Bykov R., Kerchev I., Demenkova M., Ryabinin A., Ilinsky Y. (2020). Sex-specific *Wolbachia* infection patterns in populations of *Polygraphus proximus* Blandford (Coleoptera; Curculionidae: Scolytinae). Insects.

[109] Schilthuizen M.O., Stouthamer R. (1997). Horizontal transmission of parthenogenesis–inducing microbes in *Trichogramma* wasps. Proc. R. Soc. Lond. Ser. B Biol. Sci..

[110] Huigens M.E., De Almeida R.P., Boons P.A.H., Luck R.F., Stouthamer R. (2004). Natural interspecific and intraspecific horizontal transfer of parthenogenesis–inducing *Wolbachia* in *Trichogramma* wasps. Proc. R. Soc. Lond. Ser. B Biol. Sci..

[111] Nikoh N., Hosokawa T., Moriyama M., Oshima K., Hattori M., Fukatsu T. (2014). Evolutionary origin of insect–*Wolbachia* nutritional mutualism. PNAS Nexus.

[112] Łukasik P., Guo H., Van Asch M., Ferrari J., Godfray H.C.J. (2013). Protection against a fungal pathogen conferred by the aphid facultative endosymbionts *Rickettsia* and *Spiroplasma* is expressed in multiple host genotypes and species and is not influenced by co-infection with another symbiont. J. Evol. Biol..

[113] Garcia-Arraez M.G., Masson F., Escobar J.C.P., Lemaitre B. (2019). Functional analysis of RIP toxins from the *Drosophila* endosymbiont *Spiroplasma poulsonii*. BMC Microbiol..

[114] Jaenike J., Unckless R., Cockburn S.N., Boelio L.M., Perlman S.J. (2010). Adaptation via symbiosis: Recent spread of a *Drosophila* defensive symbiont. Science.

[115] Ebbert M.A., Nault L.R. (1994). Improved overwintering ability in *Dalbulus maidis* (Homoptera: Cicadellidae) vectors infected with *Spiroplasma kunkelii* (Mycoplasmatales: Spiroplasmataceae). Environ. Entomol..

