# Supplementary material for: Endosymbiotic Bacteria Spiroplasma and Wolbachia in a Laboratory-Reared Insect Collection
Source: Insects. 2025 Nov 16;16(11):1168. doi: 10.3390/insects16111168 (PMC12653924; doi:10.3390/insects16111168)
Supplement: Supplementary file 1 [file insects-16-01168-s001.zip › Table_S1_-_Life_stages_and_DNA_extraction_sources.pdf]

Table S1. Life stages and DNA extraction sources

Species	Size	Life stage(s)	Source for DNA extraction	Symbiont
Acheta domesticus	16-21 mm	imago, eggs	abdomen	no
			eggs	no
Adalia bipunctata	5 mm	imago	abdomen	no
Aphis fabae	1-3 mm	imago/nymphs	imago/nymphs (poolled)	no
Arma custos	11-14 mm	imago, eggs	abdomen	*Spiroplasma**
			eggs	no
Brevicoryne brassicae	2,4 mm	imago/nymphs	imago/nymphs (poolled)	no
Bruchus rufimanus	3,5-5 mm	imago	whole insect	no
Chrysopa formosa 1 (origin of 2018y)	11-15 mm	imago, eggs	whole insect	*Wolbachia*
			eggs	*Wolbachia*
Chrysopa formosa 2 (origin of 2024y)	11-15 mm	imago, eggs	whole insect	*Wolbachia*
			eggs	*Wolbachia*
Coccinella septempunctata	3-7 mm	imago	abdomen	no
Grillus bimaculatus	25-40 mm	Imago	abdomen	no
Harmonia (Leis) dimidiata	6-9 mm	imago	abdomen	no
Harmonia axyridis	5-9 mm	imago	abdomen	no
Hippodamia variegata	3-6 mm	imago	abdomen	no
Lacanobia oleracea	34-44 mm	imago	abdomen	no
Macrolophus pygmaeus	2-11 mm	imago	whole insect	*Wolbachia*
Megoura viciae	4 mm	imago /nymphs	imago /nymphs (poolled)	no
Nabis ferus	8-8,5 mm	imago	whole insect	*Wolbachia*
Nabis sp.	8-8,5 mm	imago	whole insect	*Wolbachia, Spiroplasma*
Nesidiocoris tenuis	~5 mm	imago	whole insect	no
Platymeris biguttatus	10-40 mm	imago, eggs	gut	no
			gonades	
			eggs	
Plutella xylostella	9 mm	caterpillars	caterpillars (poolled)	no
Podisus maculiventris	10-14 mm	imago, eggs	whole insect	*Spiroplasma**
			eggs	no
Psyttala horrida	30-45 mm	imago, eggs	gut	no
			gonades	
			eggs	
Rhopalosiphum padi	1-3 mm	imago /nymphs	imago/nymphs (poolled)	no
Schizaphis graminum	1-2 mm	imago /nymphs	imago/nymphs (poolled)	no
Subcoccinella vigintiquatuorpunctata	3-4 mm	imago	abdomen	no
Tenebrio molitor	~10 mm	larvae	larvae	*Spiroplasma*
Trialeurodes vaporariorum	1-2 mm	imago	whole insects (poolled)	no
Trissolcus kozlovi	1-2 mm	imago	whole insects (poolled)	*Wolbachia*
Zophobas morio	~10 mm	larvae	larvae	no

Footnotes: *– occasionally false-positive signal due to contamination of a food substrate.
